# Elevated Serum Tryptase and Endothelin in Patients with ST Segment Elevation Myocardial Infarction: Preliminary Report

**DOI:** 10.1155/2015/395173

**Published:** 2015-05-18

**Authors:** Lukasz Lewicki, Janusz Siebert, Natalia Marek-Trzonkowska, Emilia Masiewicz, Tomasz Kolinski, Magdalena Reiwer-Gostomska, Radoslaw Targonski, Piotr Trzonkowski

**Affiliations:** ^1^University Center for Cardiology, Poland; ^2^Pomeranian Cardiology Centers, Jagalskiego 10, 84-200 Wejherowo, Poland; ^3^Department of Family Medicine, Medical University of Gdansk, Poland; ^4^Department of Clinical Immunology and Transplantology, Medical University of Gdansk, Poland

## Abstract

An inflammatory response plays a crucial role in myocardial damage after an acute myocardial infarction. *Objectives*. To measure serum concentrations of several mediators in patients with an acute myocardial infarction (STEMI) and to assess their potential relationship with a risk of coronary instability. *Patients and Methods*. The 33 patients with STEMI and 19 healthy volunteers were analyzed. The clinical data were obtained; as well serum concentrations of tryptase, endothelin (ET-1), angiogenin, soluble c-kit, and PDGF were measured. *Results*. Patients with STEMI had higher serum tryptase and ET-1 than healthy volunteers (2,5 ± 0,4 ng/mL versus 1,1 ± 0,4 ng/mL and 0,7 ± 0,1 ng/mL versus 0,3 ± 0,1 ng/mL, resp.). Subjects with significant lesion in left anterior descending artery (LAD) had lower serum ET-1 compared to those with normal LAD (0,6 ± 0,2 pg/mL versus 0,9 ± 0,4 pg/mL). Patients with three-vessel coronary artery disease (CAD) had higher level of soluble c-kit compared to those with one- or two-vessel CAD: 19,9 ± 24,1 ng/mL versus 5,6 ± 1,9 ng/mL. *Conclusions*. Elevated serum tryptase and ET-1 may be markers of increased coronary instability; some cytokines may be related to the extension of CAD.

## 1. Introduction

The pathomechanism of coronary artery disease (CAD) is heterogeneous and complex. CAD remains a leading cause of morbidity and mortality among patients. Investigating and understanding its predictors can be a great step forward in disease prevention and treatment. An acute myocardial ischemia provokes an inflammatory response that increases myocardial damage, but on the other hand it promotes regenerative process. This process is a consequence of endothelial progenitor cell mobilization, which is observed after an acute myocardial infarction.

Tryptase is a serine protease present in all mast cells, also in the mast cells of human atherosclerotic lesions [1]. Tryptase is released from activated mast cells and once released into the extracellular fluid can exert many pathophysiologically relevant actions, which tend to promote plaque development. Thus, the released tryptase degrades high-density lipoproteins (HDL), and by blocking HDL-dependent cholesterol efflux from foam cells is involved in foam cell formation [2]. This way tryptase may promote lipid accumulation during the initiation of atherosclerosis. Extracellular tryptase can also activate matrix-degrading metalloproteinases, notably stromelysin (MMP3), which can then activate other MMPs in the plaque [3]. This ability of tryptase could be a significant factor, which contributes to plaque destabilization, and ultimately result in plaque rupture [4]. Moreover, mast cells play a role in the late phases of atherogenesis by releasing proinflammatory cytokines, such as TNF-alpha, which can induce, for example, the production of MMP-9 in macrophages [5, 6]. They circulate in blood as indeterminate progenitor cells, and when they migrate from the circulation into tissue, they differentiate into mature mast cells. Mast cells accumulate at sites of inflammation and when activated they play a role in wound healing, tissue fibrosis, and angiogenesis [7]. Importantly, increased numbers of activated mast cells are found in the culprit coronary lesions of patients with myocardial infarction, which likely reflects their ability to secrete proteases and activate other proteases in the advance inflamed coronary plaques [4]. SCF (stem cell factor) is a chemotactic agent for mast cells, bone marrow stem cells (BMSC), progenitor cells, and hematopoietic stem cells. Its role is the mediation of cell adhesion by binding directly to c-kit located on BMSC and enabling cell attachment. What is more, SCF c-kit mediated signalling plays an important role in cardiac stem cell differentiation and recruitment of endothelial progenitor cells during inflammation [8]. Kit, SCF receptor, is normally present in both cell surface and soluble forms. Soluble c-kit modulates SCF activity by blocking the ability of SCF to stimulate different cells growth [7].

Other mediators may play a role in pathology of CAD.

Endothelin-1 (ET-1) is a potent vasoconstrictor, proinflammatory mediator, and mitogen produced in response to hypoxia and vessel wall stress. It is known to play a role in endothelial dysfunction and inflammation and can contribute to atherosclerotic plaque formation [9]. Angiogenin is a growth factor with a potent function in creating new blood vessels. Physiologically, angiogenin is induced during inflammation and exhibits wound healing properties. It is also required for other angiogenic factors to stimulate angiogenesis [10, 11]. Platelet-derived growth factor (PDGF) plays an important role in stimulating growth, survival, and motility of mesenchymal cells and certain other cell types. PDGF is also crucial during embryonic development and in the control of tissue homeostasis in the adult [12].

Taking into account the role of all these mediators in development and pathology of CAD we decided to measure their serum concentration in patients with an acute ST segment elevation myocardial infarction (STEMI) and healthy volunteers in order to assess the potential biochemical risk factors of an acute coronary syndrome.

## 2. Materials and Methods

This was a prospective and single-center study. The study protocol was approved by the local ethics committee. All patients gave written informed consent.

### 2.1. Patients

Between November 2012 and May 2013, we prospectively screened 122 consecutive patients who underwent primary percutaneous coronary intervention (pPCI) because of ST elevation myocardial infarction (STEMI) in the Department of Invasive Cardiology, Pomeranian Cardiology Centers, Wejherowo, Poland. Patients with renal failure, malignancy, and acute or chronic inflammatory disease were excluded from the study. Finally, 33 patients were included. The complete demographic and clinical data were obtained. These patients were compared to a control group of 19 healthy volunteers. These control subjects were inhabitants of Pomeranian region; all of them were nonsmokers and they were carried by one general physician's practice.

### 2.2. Percutaneous Coronary Intervention

All STEMI patients underwent pPCI by transradial or transfemoral artery approach. A loading dose of 300 mg of Aspirin and 600 mg of Clopidogrel combined with a bolus of 60 IU/kg of unfractionated heparin were administered to the patients. The decision on use of IIb/IIIa inhibitors, thrombectomy device, direct stenting, or balloon angioplasty was left to the interventional cardiologist performing the procedure.

### 2.3. Blood Sampling and Laboratory Tests

The blood samples were obtained after puncturing of a radial or a femoral artery and they were drawn from the vascular sheath during a coronary angiography.

Then, blood samples were centrifuged at 1000 ×g for 10 minutes to obtain serum. Subsequently, standard clinical parameters were measured and the remaining serum was apportioned into 0.5 mL aliquots and stored at −80°C until analysis of tryptase, PDGF, soluble c-kit, endothelin, and angiogenin.

### 2.4. Measurement of Tryptase, PDGF, Soluble c-kit, Endothelin, and Angiogenin

Serum concentrations of studied mediators were measured with ELISA kits according to the manufacturer's instructions and analyzed with multilabel plate reader VICTOR X4 (Perkin Elmer). ELISA kit for measurement of tryptase was purchased from USCN Life Science Inc., while PDGF, soluble c-kit, endothelin, and angiogenin were measured with ELISA Quantikine tests from R&D Systems.

### 2.5. Statistical Analysis

The results are expressed as mean ± SD. Comparisons between groups were performed using Student's *t*-test and Kolmogorov-Smirnov test for continuous variables. The levels of tryptase, endothelin-1 (ET-1), PDGF, soluble c-kit, and angiogenin were adjusted for age, gender, BMI, lipid profile, and LVEF using a linear regression model. Statistica 10.0 Statsoft version and Wizard Statistics 1.6.2 version were used for analysis and *p* value <0, 05 was considered statistically significant.

## 3. Results and Discussion

### 3.1. Results

The clinical data of 33 patients with STEMI are presented in Table 1.

Patients with STEMI did not differ from healthy group according to age, levels of total cholesterol, LDL, and triglycerides. We noticed significantly higher level of CRP in STEMI patients compared to healthy volunteers. Overweight expressed by body mass index (BMI) was significantly higher in STEMI patients than in the control group. There was a significantly higher level of creatinine in patients with acute myocardial infarction; however mean GFR calculated according to MDRD formula was 57,8 mL/min/1,73 m^2^ body surface area (BSA). This parameter was unavailable for healthy volunteers. Subjects with an acute myocardial infarction had significantly higher level of HbA1c; in contrast, they presented with lower level of HDL compared to healthy group (Table 2).

Angiographic data of 33 STEMI patients are presented in Table 3. More than twenty percent of STEMI patients presented with three-vessel coronary artery disease. An inferior acute myocardial infarction was the most common diagnosis on admission in patients admitted for STEMI. In most of the cases a culprit lesion was a right coronary artery.

In linear regression model, serum tryptase levels were associated significantly with age. The soluble c-kit concentration corresponded with BMI and LVEF. In turn, there was a significant inverse correlation of angiogenin with total cholesterol and LDL (Table 4).

**Figure 1:**
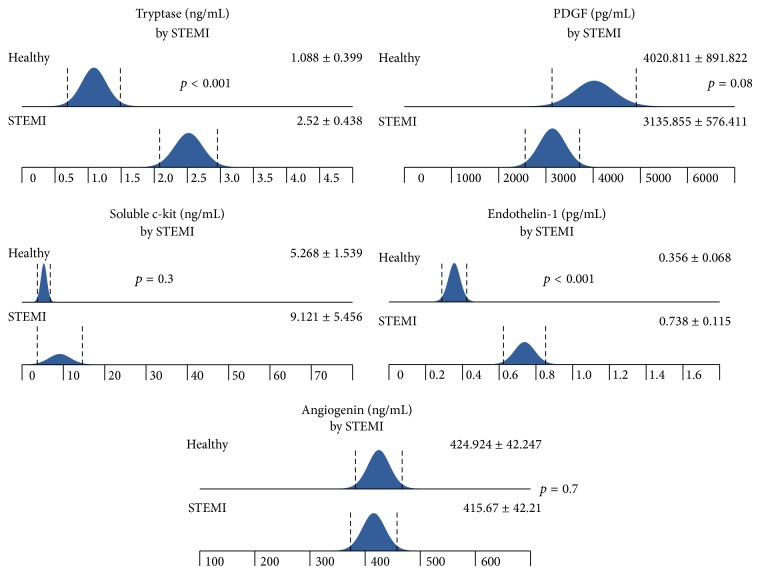
The differences in tryptase, PDGF, soluble c-kit, ET-1, and angiogenin levels between STEMI and healthy groups. The analysis was made using *t*-test.

Patients with STEMI had significantly higher level of tryptase and ET-1 on admission than subjects from healthy group. There was an insignificant trend to lower level of PDGF in patients with STEMI compared to healthy volunteers (Figure 1).

The STEMI patients with history of diabetes had significantly higher level of soluble c-kit compared with patients without diabetes (Table 5).

These same findings were observed in patients with reduced ejection fraction heart failure (REF-HF) and in patients with family history of coronary artery disease (CAD) (Tables 6 and 7).

**Figure 2:**
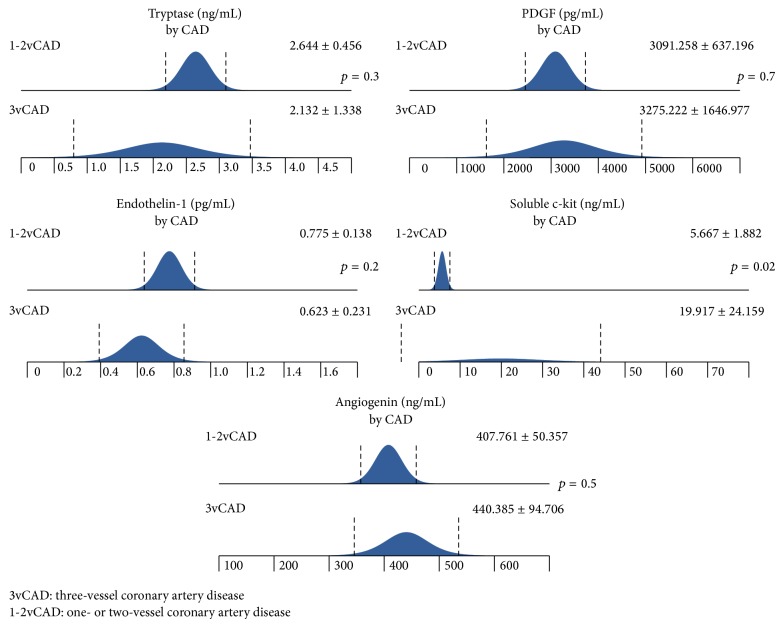
The differences in tryptase, PDGF, soluble c-kit, ET-1, and angiogenin levels among patients with 1-, 2-, and 3-vessel CAD. The analysis was made using *t*-test.

There was a significantly higher level of soluble c-kit among patients with three-vessel coronary artery disease (CAD) compared to those with two- or one-vessel CAD (Figure 2).

**Figure 3:**
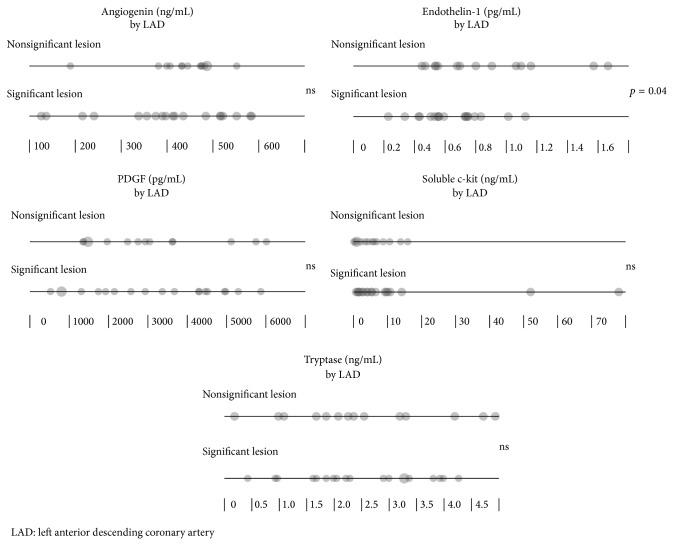
The differences in tryptase, PDGF, soluble c-kit, ET-1, and angiogenin levels between STEMI patients with or without significant LAD stenosis.

Patients with significant lesion in left anterior descending coronary artery (LAD) had lower level of ET-1 compared to those of normal LAD (Figure 3 and Table 8).

### 3.2. Discussion

In this preliminary study we found elevated levels of tryptase and endothelin-1 in patients with STEMI compared to healthy volunteers. These observations correspond to observations of Xiang et al. who found that an elevated level of tryptase is independent marker of coronary plaque instability [13]. What is more, Chen et al. investigated that high tryptase levels after PCI were associated with poor myocardial reperfusion and poor cardiac function [14]. However, Pastorello et al. observed that the acute tryptase increase did not strictly identify patients with acute myocardial infarction and its level was related to the severity of the clinical course [15]. In our study, patients with STEMI had twofold higher level of tryptase than healthy volunteers. We noticed significant positive correlation of serum tryptase and age, which is also consistent with previous data [13, 16].

Endothelin-1 is responsible for endothelial dysfunction and inflammation and contributes to atherosclerotic plaque formation. In the acute phase of MI, ET-1 enhances myocardial necrosis and arrhythmias. However, it seems to exert a positive effect on further infarct healing and early ventricular remodeling. Later in postinfarction phase, ET-1 leads to left ventricular afterload increase and participates in fibrotic process of the myocardium [17, 18]. In our study we found a twofold higher level of ET-1 in STEMI patients compared to the healthy control group. Additionally, we noticed a reduced level of ET-1 in patients with significant stenosis in LAD coronary artery compared to those without that lesion.

We have shown the higher level of soluble c-kit among patients with diabetes, family history of CAD, and REF-HF. It is very interesting that, in patients with heart failure, concentration of soluble c-kit is higher compared to patients with preserved left ventricle ejection fraction, because when transmembrane form of SCF binds to soluble c-kit there is no signal transduction and no survival or proliferation signal is delivered to mast cells. On the other hand, Hara et al. reported that cardiac mast cells seem to be involved in the transition from compensated hypertrophy to decompensated hypertrophy and heart failure [19]. Increase in concentration of soluble c-kit might reflect the protectory process in heart failure. The results of our research oblige us to continue the investigation in order to explain it.

There was an inversed correlation of soluble c-kit level with LVEF and BMI in the linear regression model. We did not find any correlation of soluble c-kit level with cholesterol nor other biochemical parameters among STEMI patients. Very few data concerning the behavior of soluble c-kit in context of coronary heart disease could be found. Mast cells can be found in heart tissue, and several authors suggested that myocardial mast cells seem to be involved in the pathogenesis of ischemia [7]. However, the pathomechanism is still unclear.

Angiogenin, a very potent angiogenic factor, also plays a crucial role in cell proliferation. Our previous studies and the studies of the others showed that angiogenin not only stimulates angiogenesis but also exhibits anti-inflammatory and immunosuppressive activity [10, 11, 20, 21] and its levels are decreased in inflammatory environment, such as that observed in poorly controlled diabetes and diabetic vascular complications [20, 21]. Nevertheless, proangiogenic activity of this mediator may have also a deleterious impact on CAD. Angiogenin can stimulate plaque vascularization and thus lead to plaque destabilization. This hypothesis seems to be supported by study of Tello-Montoliu et al. who suggested that angiogenin might be a marker of unstable plaque during the acute event and a risk marker of future events [11].

Liu et al. tested the hypothesis that PDGF contributes to cardiac angiogenesis and fibro genesis after myocardial infarction. These investigations indicated that PDGF can play a role in the development of cardiac interstitial fibrosis in the noninfarcted myocardium but does not affect scar formation in the infarcted myocardium [22].

We did not find any significant differences in selected angiogenic factors such as PDGF and angiogenin among patients with STEMI compared to healthy controls. However, we noticed a significantly higher level of C-reactive protein in subjects with an acute MI, which seems to be the common finding among patients with an acute coronary syndrome. We observed significant inverse correlation of angiogenin levels and total cholesterol and LDL.

Although no significant observations of the levels of angiogenic growth factors between diabetic and nondiabetic STEMI patients could be reported, our previous research indicates that diabetes strongly influences the process of angiogenesis [21, 23]. The number of evaluated diabetic patients was small; that is why further research might reveal more interesting facts.

The two very interesting findings should also be mentioned: a positive correlation of serum soluble c-kit and three-vessel coronary artery disease and the lower level of ET-1 in patients with significant stenosis in LAD. According to our knowledge, there are limited data on correlation of these cytokines and extension of CAD.

Although our preliminary report is limited because of relatively small study sample, we believe that it may be an interesting introduction to further investigations concerning larger group of patients with different clinical manifestations of coronary artery disease and we hope to present these results in the near future.

## 4. Conclusions

1. Elevated serum tryptase and ET-1 may be markers of increased coronary instability; moreover, some cytokines may be related to the extension of CAD.
2. An elevated level of soluble c-kit in patients with heart failure may reflect the presence of protective process in a severely diseased heart.

## Figures and Tables

**Table 1:** The clinical data of 33 patients with STEMI.

STEMI anterior	15 (45,5%)
STEMI inferior	17 (51,5%)
STEMI lateral	1 (3%)
History of previous MI	1 (3%)
Family history of CAD	8 (24,2%)
Hypertension	13 (39,4%)
Diabetes	5 (15,2%)
LVEF [%]	43,8 ± 9,6
REF-heart failure	5 (15,2%)
3vCAD	8 (24,2%)

BMI: body mass index; STEMI: ST segment elevation myocardial infarction; MI: myocardial infarction; CAD: coronary artery disease; LVEF: left ventricle ejection fraction; REF: reduced ejection fraction; 3vCAD: three-vessel coronary artery disease.

**Table 2:** The comparison of demographic and biochemical data of 33 STEMI patients and 19 healthy volunteers. The analysis was made using *t*-test and Kolmogorov-Smirnov test.

	STEMI	Healthy volunteers	*p* value
Age [years]	64,4 ± 3,8	60,1 ± 9,1	0,3
Gender [male/female]	23/10	6/13	0,01
History of smoking	19 (57,6%)	None	—
BMI [kg/m^2^]	27,5 ± 1,6	24,5 ± 1,4	0,01
CRP [mg/L]	26,8 ± 20,3	1,6 ± 0,8	0,01
Creatinine level [mg/dL]	1,0 ± 0,1	0,8 ± 0,1	0,02
eGFR [mL/1,73 m^2^ BSA]	57,8 ± 2,9	unavailable	—
Total cholesterol [mg/dL]	205,4 ± 17,1	226,5 ± 18,8	0,1
HDL [mg/dL]	44,6 ± 4,1	61,0 ± 8,4	<0,001
Triglycerides [mg/dL]	128,1 ± 37,5	98,1 ± 17,0	0,2
LDL [mg/dL]	140,0 ± 14,3	146,4 ± 16,8	0,6
HbA1c [%]	7,2 ± 1,4	5,8 ± 0,2	<0,001

BMI: body mass index; CRP: C-reactive protein; GFR: glomerular filtration rate.

**Table 3:** Angiographic data of 33 STEMI patients.

	LM	LAD	CX	RCA
Significant stenosis	3 (9,1%)	19 (57,6%)	13 (39,4%)	23 (69,7%)
Culprit lesion	1 (3%)	14 (42,4%)	3 (9,1%)	15 (45,5%)

LM: left main coronary artery; LAD: left anterior descending coronary artery; CX: circumflex coronary artery; RCA: right coronary artery.

**Table 4:** The linear regression model of correlation between demographic and biochemical data in 33 STEMI patients. The analysis was made using linear regression model.

	Correlation coefficient	*p* value
Tryptase with age	0,9	0,03
Soluble c-kit with BMI	−0,4	0,04
Soluble c-kit with LVEF	−0,5	0,004
Angiogenin with TCHOL	−0,5	0,01
Angiogenin with LDL	−0,4	0,03

BMI: body mass index; LVEF: left ventricle ejection fraction.

**Table 5:** The differences in tryptase, PDGF, soluble c-kit, ET-1, and angiogenin levels between diabetic and nondiabetic STEMI patients. The analysis was made using *t*-test.

	Diabetes	No diabetes	*p* value
Tryptase [ng/mL]	1,9 ± 1,2	2,6 ± 1,2	0,2
PDGF [pg/mL]	4126,1 ± 608,6	2959 ± 1692	0,1
Soluble c-kit [ng/mL]	23,7 ± 30,4	6,5 ± 9,8	0,02
Endothelin [pg/mL]	0,6 ± 0,2	0,7 ± 0,3	0,4
Angiogenin [ng/mL]	412,5 ± 105,3	416,2 ± 123,1	0,9

**Table 6:** The differences in tryptase, PDGF, soluble c-kit, ET-1, and angiogenin levels between REF-HF and no REF-HF STEMI patients. The analysis was made using *t*-test.

	REF-HF	No REF-HF	*p* value
Tryptase [ng/mL]	1,9 ± 1,2	2,6 ± 1,2	0,2
PDGF [pg/mL]	2900 ± 1576,2	3112,4 ± 1653	0,8
Soluble c-kit [ng/mL]	21,4 ± 32	5,2 ± 4,2	0,01
Endothelin [pg/mL]	0,7 ± 0,2	0,7 ± 0,3	0,7
Angiogenin [ng/mL]	356,8 ± 177,2	422,6 ± 107	0,2

REF-HF: reduced ejection fraction heart failure.

**Table 7:** The differences in tryptase, PDGF, soluble c-kit, ET-1, and angiogenin levels between STEMI patients with or without family history of CAD. The analysis was made using *t*-test.

	Family history of CAD	No family history of CAD	*p* value
Tryptase [ng/mL]	2,3 ± 1,5	2,5 ± 1,2	0,7
PDGF [pg/mL]	3178,5 ± 1752,2	2991,1 ± 1622,3	0,8
Soluble c-kit [ng/mL]	16,1 ± 25,1	4,6 ± 4,3	0,04
Endothelin [pg/mL]	0,7 ± 0,2	0,8 ± 0,3	0,9
Angiogenin [ng/mL]	405,5 ± 122,7	422,2 ± 117,6	0,7

CAD: coronary artery disease.

**Table 8:** The differences in tryptase, PDGF, soluble c-kit, ET-1, and angiogenin levels between STEMI patients with or without significant LAD stenosis. The analysis was made using *t*-test.

	Significant stenosis of LAD	No significant stenosis of LAD	*p* value
Tryptase [ng/mL]	2,5 ± 1,1	2,5 ± 1,4	0,9
PDGF [pg/mL]	3184 ± 1686,8	3070,5 ± 1599,1	0,8
Soluble c-kit [ng/mL]	11,6 ± 19,6	5,7 ± 5	0,3
Endothelin [pg/mL]	0,6 ± 0,2	0,9 ± 0,4	0,04
Angiogenin [ng/mL]	399,8 ± 139,6	437,2 ± 83,8	0,4

LAD: left anterior descending coronary artery.
